# Ocimum micranthum essential oil as a sustainable phytotherapeutic alternative against Haemonchus contortus

**DOI:** 10.1590/S1984-296120260011

**Published:** 2026-05-25

**Authors:** Andreza Pereira Braga, Matheus Luiggi Freitas Barbosa, Raphael Ferreira Oliveira, Karin Vitória Maia Mendonça Ferreira, Sara Stefane da Silva Cardoso, Rayane de Araújo Souza, Rita de Cássia Alves Pereira, Francisco Flávio da Silva Lopes, Gracielle Araújo Frota, Selene Maia de Morais, Letícia Oliveira da Rocha, Wesley Lyeverton Correia Ribeiro, Lorena Mayana Beserra de Oliveira

**Affiliations:** 1 Universidade Estadual do Ceará – UECE, Faculdade de Veterinária, Laboratório de Doenças Parasitárias, Programa de Pós-graduação em Ciências Veterinárias – PPGCV, Fortaleza, CE, Brasil; 2 Embrapa Agroindústria Tropical, Fortaleza, CE, Brasil; 3 Universidade Estadual do Ceará – UECE, Laboratório de Química de Produtos Naturais, Programa de Pós-graduação em Ciências Veterinárias – PPGCV, Fortaleza, CE, Brasil; 4 Universidade Estadual do Norte Fluminense Darcy Ribeiro – UENF, Centro de Biociências e Biotecnologia, Laboratório de Biologia Celular e Tecidual, Campos dos Goytacazes, RJ, Brasil; 5 Universidade Federal do Ceará – UFC, Faculdade de Medicina, Departamento de Fisiologia e Farmacologia, Fortaleza, CE, Brasil

**Keywords:** Anthelmintic activity, *Ocimum campechianum* Mill., basil, phytotherapy, gastrointestinal nematodes, small ruminants, Atividade anti-helmíntica, *Ocimum campechianum* Mill., manjericão, fitoterapia, nematódeos gastrintestinais, pequenos ruminantes

## Abstract

This study is the first to evaluate the anthelmintic activity of *Ocimum micranthum* essential oil against *Haemonchus contortus*. The oil was chemically characterized using gas chromatography–mass spectrometry. Anthelmintic activity was assessed using the egg hatching test (EHT), larval development test (LDT), larval migration inhibition test (LMIT), and adult worm motility test (AWMT) against a multidrug-resistant *H. contortus* isolate. Following AWMT, oil-induced morphological and ultrastructural alterations were investigated using scanning electron microscopy (SEM) and transmission electron microscopy (TEM). Eugenol was the major oil component (78.44%). The oil exhibited anthelmintic activity in all assays, with EC_50_ values of 0.19 (EHT), 0.031 (LDT), 0.63 (LMIT) and 0.046 (AWMT) mg/mL. At 0.25 mg/mL, complete inhibition of adult motility was observed at the first evaluation time point (3 h post-exposure). SEM revealed damage to the cuticle, buccal capsule, and vulvar flap, whereas TEM demonstrated alterations in internal tissues. *Ocimum micranthum* essential oil was effective against *H. contortus* eggs, larvae, and adults. Therefore, further studies evaluating its toxicological safety and efficacy in small ruminants are required to validate its potential as an anthelmintic.

## Resumo

Este estudo é o primeiro a avaliar a atividade anti-helmíntica do óleo essencial de *Ocimum micranthum* contra *Haemonchus contortus.* O óleo foi caracterizado quimicamente por cromatografia gasosa acoplada à espectrometria de massas. A atividade anti-helmíntica foi avaliada utilizando os testes de eclosão de ovos (TEO), desenvolvimento larvar (TDL), inibição da migração larvar (TIML) e motilidade de nematódeos adultos (TMA) contra um isolado de *H. contortus* multirresistente. Após o TMA, alterações morfológicas e ultraestruturais induzidas pelo óleo foram investigadas por microscopia eletrônica de varredura (MEV) e transmissão (MET). O eugenol foi o principal componente do óleo (78,44%). O óleo exibiu atividade anti-helmíntica em todos os ensaios, com valores de CE_50_ de 0,19 (TEO), 0,031 (TDL), 0,63 (TIML) e 0,046 (TMA) mg/mL. Na concentração de 0,25 mg/mL, observou-se inibição completa da motilidade dos adultos no primeiro ponto de avaliação (3 h após exposição). A MEV revelou danos à cutícula, à cápsula bucal e à aba vulvar, enquanto a MET demonstrou alterações nos tecidos internos. O óleo essencial de *O. micranthum* mostrou-se eficaz contra ovos, larvas e adultos de *H. contortus*. Portanto, estudos adicionais avaliando sua segurança toxicológica e eficácia em pequenos ruminantes são necessários para validar seu potencial como anti-helmíntico.

## Introduction

Gastrointestinal nematode (GIN) parasitism represents a major constraint to the welfare and productivity of small ruminants, leading to substantial economic losses because of reduced animal performance and the costs associated with anthelmintic treatments (Maestrini et al., 2022). Among these parasites, *Haemonchus contortus* is considered the most pathogenic species infecting sheep and goats owing to its hematophagous behavior, which can lead to severe anemia and, in some cases, death (Arsenopoulos et al., 2021).

Control of GIN infections has traditionally relied on the use of synthetic anthelmintics (Kaplan, 2004). However, decades of intensive or often inappropriate use have led to widespread anthelmintic resistance. This resistance reduces treatment efficacy, allowing parasite populations to persist despite chemical interventions (Arango-De La Pava et al., 2024). Moreover, anthelmintic residues can contaminate the environment via excretion into soil and may persist in animal-derived products. Furthermore, increasing consumer awareness has intensified the demand for organic products (Maestrini et al., 2022). Consequently, greater emphasis has been placed on sustainable parasite control, and alternative strategies have been explored (Kļaviņa et al., 2023).

Natural products, such as essential oils (EOs) extracted from plants, may provide an alternative strategy to reduce the reliance on synthetic anthelmintics in sheep and goat production systems (Braga et al., 2025). Several EOs have been evaluated for their effectiveness against GIN in small ruminants and have demonstrated activity both *in vitro* and *in vivo* (Araújo-Filho et al., 2019; Barbosa et al., 2023; Amel et al., 2024). Furthermore, some EOs have shown efficacy against *H. contortus* isolates resistant to synthetic compounds (Araújo-Filho et al., 2018; Barbosa et al., 2023).

The plant species *Ocimum micranthum* Willd. (syn. *Ocimum campechianum* Mill.), belonging to the Lamiaceae family, is native to the tropical regions of South and Central America and is commonly known as wild Amazonian basil, alfavaca-de-monte, alfavaca-de-campo, or alfavaca-silvestre (Tacchini et al., 2021; Wilson et al., 2022). This species has a broad geographical distribution and grows spontaneously in coastal vegetation and tropical forests, adapting well to tropical climates, with an annual growth cycle, and develops in permeable soils. It is also commonly found in domestic gardens, cultivated fields, and along roadsides (Wilson et al., 2022; Calvo‐Irabien, 2025).

The chemical EO obtained from its leaves has been reported to contain eugenol, β-elemene, γ-elemene, β-caryophyllene, isoeugenol, and methyl eugenol as major constituents (Silva et al., 1998; Caamal-Herrera et al., 2016). EOs from other *Ocimum* species with similar chemical profiles, such as *O. gratissimum* and *O. basilicum*, have demonstrated inhibitory effects on *H. contortus* egg hatching (Pessoa et al., 2002; Sousa et al., 2021).

Previous studies have reported the insecticidal (Scalvenzi et al., 2019), antifungal (Vieira et al., 2014; Caamal-Herrera et al., 2018), antinociceptive (Lino et al., 2005), and analgesic (Sacchetti et al., 2004) properties of *O. micranthum* EO. However, its anthelmintic activity has not yet been described. Therefore, the present study aimed to evaluate the anthelmintic effect of *O. micranthum* EO against the eggs, larvae, and adults of a multidrug-resistant *H. contortus* isolate and to investigate oil-induced morphological and ultrastructural alterations in adult parasites.

## Material and methods

### EO extraction and chemical analysis

The aerial parts (fresh leaves and flowers) of *O. micranthum* were collected in May 2024, during the rainy season, from the medicinal plant garden of Embrapa Agroindústria Tropical (3°45’5”S, 38°34’37’’W). A voucher specimen was deposited at the Prisco Bezerra Herbarium of the Federal University of Ceará (Protocol number: EAC 56852).

The EO was obtained via hydrodistillation using a Clevenger-type apparatus. Chemical characterization was performed using gas chromatography-mass spectrometry (GC–MS) using a GCMS-QP2010S (Shimadzu^®^, Japan). Analyses were conducted under the following conditions: Rtx-5MS capillary column (30 m × 0.25 mm × 0.25 µm df); helium as carrier gas at constant linear velocity (24.2 mL/min); injector temperature of 250 °C; split ratio of 1:100; column temperature programmed from 35–180 °C at 4 °C/min, then from 180–280 °C at 17 °C/min, with a final hold at 280 °C for 10 min; and detector temperature of 250 °C. The total run time was 55 min.

Sample (1 µL) was injected at a concentration of 1 mg/mL. For MS, electron impact ionization was performed at 70 eV. Compounds were identified by comparing relative retention indices (Adams, 2007) with spectra available in the National Institute of Standards and Technology database.

### Anthelmintic activity

*Haemonchus contortus* Kokstad isolate, known to be resistant to benzimidazoles, levamisole and macrocyclic lactones (Neveu et al., 2007; Fauvin et al., 2010), was used as the reference strain. The isolate was provided by the Institut National de Recherche pour l'Agriculture, l'Alimentation et l'Environnement (INRAE), Nouzilly, France.

This study was approved by the Ethics Committee for the Use of Animals (Protocol number: 31032.004466/2025-05) of the Universidade Estadual do Ceará, Brazil. A lamb was housed in metabolic cages and treated with ivermectin (0.2 mg/kg; Ivermic^®^, Microsules, Brazil), levamisole (5 mg/kg; Ripercol^®^, Zoetis, Brazil), albendazole (10 mg/kg; Albendathor 10^®^, JA Saúde Animal, Brazil), and monepantel (2.5 mg/kg; Zolvix^®^, Elanco, Brazil), administrated as single doses on alternate days. After confirmation of complete clearance of the natural infection using fecal egg counts (epg) and coprocultures, the animal was orally infected with 10,000 third-stage larvae (L3) of *H. contortus*. This animal served as the source of eggs, larvae, and adult worms for all *in vitro* assays. Experimental infection was monitored weekly using epg and biweekly using fecal cultures.

### Egg hatching test (EHT)

*Haemonchus contortus* eggs were recovered from feces collected directly from the rectum of monospecifically infected animal using the technique described by Hubert & Kerboeuf (1992). EHT was performed according to the method described by Coles et al. (2006). Aliquots of 250 μL containing approximately 100 freshly recovered eggs were incubated for 48 h at 25 °C with 250 μL of *O. micranthum* EO diluted in 0.5% DMSO at concentrations of 0.06, 0.125, 0.25, 0.5 and 1 mg/mL. After incubation, egg hatching was halted by adding drops of 5% Lugol’s solution. Eggs and first-stage larvae (L1) were then counted under a light microscope. Negative (0.5% DMSO) and positive (0.025 mg/mL thiabendazole) controls were included. Each treatment and control group consisted of five replicates, and three independent experiments.

### Larval development test (LDT)

Following egg recovery using the technique described by Hubert & Kerboeuf (1992), LDT was performed according to methodology by Coles et al. (2006) with modifications. Egg suspension was incubated for 24 h at 27 ± 1 °C to obtain L1 larvae. Subsequently, 170 μL of L1 suspension (≈ 200 larvae) and 80 μL of nutrient medium (lyophilized *Escherichia coli*, yeast extract, and amphotericin B) were incubated in 24-well plates with 250 μL of *O. micranthum* EO at concentrations of 0.007, 0.015, 0.03, 0.06 and 0.12 mg/mL. Plates were homogenized and incubated for an additional 6 days at 27 ± 1 °C under optimal humidity (>80%). After incubation, L3 were counted using an inverted microscope. DMSO (0.5%) and ivermectin (0.008 mg/mL) solutions were used as negative and positive controls, respectively. Five replicates were used for each treatment and control.

### Larval migration inhibition test (LMIT)

L3 were recovered according to the technique described by Roberts and O’Sullivan (1950). LMIT was conducted following protocols adapted from Bortoluzzi et al. (2021) and Demeler et al. (2010). Larvae were unsheathed using sodium hypochlorite containing 2.0–2.5% active chlorine (1 µL sodium hypochlorite per 200 µL of L3 suspension) for 1 h under magnetic stirring. Larvae were then centrifugated thrice (5 min at 2,000 rpm) with phosphate-buffered saline (PBS). Aliquots of 400 μL containing ≈ 200 unsheathed larvae/tube were then incubated at 27 ± 1 °C for 18 h (first incubation) with 400 μL of *O. micranthum* EO at concentrations of 0.25, 0.50, 1, 2 and 4 mg/mL. After incubation, the suspensions were transferred to 24-well plates fitted with containing apparatus fitted 25 µm mesh and incubated for an additional 24 h under the same conditions (second incubation). The apparatus was then removed, and the migrated larvae were counted using an inverted microscope. Negative (0.5% DMSO) and positive (0.25 mg/mL ivermectin) controls were included. Each treatment consisted of five replicates, and three independent experiments were performed.

### Adult worm motility test (AWMT)

AWMT was performed following the methodology described by Hounzangbe-Adote et al. (2005). Experimentally infected lamb was euthanized using chemical methods. For this, the sedation was followed by general anesthesia. After confirmation of unconsciousness and loss of reflex, potassium chloride was administered. Immediately after euthanasia, the abomasum was removed, opened, and placed in PBS at 37 °C. Adult *H. contortus* females were collected and transferred to 24-well plates (three specimens per well) containing 500 μL of PBS supplemented with 4% penicillin–streptomycin (Sigma-Aldrich^®^) and incubated for 1 h at 37 °C under 5% CO_2._ Subsequently, 500 μL aliquots of *O. micranthum* EO at concentrations of 0.015, 0.03, 0.06, 0.12, and 0.25 mg/mL were added to each well. Worm motility was assessed after 3, 6, 9, and 12 h of incubation under the same conditions under a stereomicroscope. Negative (0.5% DMSO) and positive (0.10 mg/mL ivermectin) controls were included. Five replicates were used per treatment and control.

### Scanning electron microscopy (SEM) and transmission electron microscopy (TEM)

Adult *H. contortus* specimens obtained from the AWMT after 12 h of exposure to EO (0.25 mg/mL), negative control (DMSO), and positive control (ivermectin) were analyzed using SEM and TEM following methodology adapted from Rocha et al. (2020). Worms were fixed in 2.5% glutaraldehyde and 4% paraformaldehyde in 0.1 M sodium cacodylate buffer (pH 7.1) for 48 h. For SEM, samples were washed thrice in buffer, dehydrated in a graded ethanol series (30, 60, 70, 80, 90 and 100%, 5 min each), dried using an EMSCOPE CPD 750, mounted on metal stubs, and sputter-coated with gold–palladium for 5 min at 100 Å min^−1^. Observations in the parasites’ cuticle were performed using a JEOL JSM-IT210 scanning electron microscope (JEOL Ltd., Tokyo, Japan) at 20 kV. For TEM, the specimens were dehydrated in graded acetone, infiltrated with Spurr resin, and polymerized at 60 °C. Ultrathin sections (70 nm) were contrasted with uranyl acetate and lead citrate and examined using a JEOL JEM 1400 Plus (JEOL Ltd., Tokyo, Japan) transmission electron microscope at 80 kV.

### Statistical analysis

In the EHT, inhibition of egg hatching was calculated as: [number of eggs / (number of eggs + number of L1)] ×100. In LDT and LMIT, inhibition was calculated as: [(mean number of L3 in the negative control – number of L3 in the treated group) / mean number of L3 in the negative control] ×100. In the AWMT, motility inhibition was expressed as the percentage of motionless worms relative to the total number of worms per well.

Data were analyzed via analysis of variance (ANOVA) using GraphPad Prism^®^ version 8.0.1. Results from EHT, LDT, and LMIT were analyzed using one-way ANOVA, whereas AWMT data were analyzed using two-way repeated-measures ANOVA, followed by Tukey’s post hoc test. Effective concentration required to inhibit 50% (EC_50_) of egg hatching, larval development, larval migration, and adult motility were estimated via probit regression using SPSS^®^ version 23.0 for Windows.

## Results

The chemical composition of *O. micranthum* EO is shown in Table 1. GC–MS analysis identified eugenol as the predominant constituent (78.44%). Nineteen additional compounds were detected, with β-caryophyllene (5.10%), elixene (4.26%) and elemicin (2.70%) being the most abundant among the minor constituents.

**Table 1:** Composition of *Ocimum micranthum* essential oil, as determined using gas chromatography–mass spectrometry.

**Constituents**	**Ri**	**Percentage (%)**
Eucalyptol	1025	0.50
β-Ocimene	1035	0.08
Linalool	1100	0.52
Borneol	1169	0.08
Eugenol	1369	78.44
β-Elemene	1397	1.12
Methyl eugenol	1411	1.38
β-caryophyllene	1423	5.10
α-humulene	1454	1.00
Alloaromadendrene	1461	0.23
γ-Muurolene	1480	0.21
β-Selinene	1484	0.92
Elixene	1494	4.26
δ-Guaiene	1502	1.48
β-Bisabolene	1505	0.57
Elemicin	1549	2.70
Spathulenol	1566	0.33
Caryophyllene oxide	1570	0.77
Globulol	1578	0.11
Selin-7(11)-en-4-ol	1631	0.21

Ri: Retention index.

The effects of *O. micranthum* EO on *H. contortus* egg hatching are shown in Table 2. The oil exhibited ovicidal activity at all tested concentrations, with a concentration-dependent response. Complete inhibition of egg hatching (100%) was observed at the highest concentration tested, whereas 98.01% inhibition was recorded at the second-highest concentration. These values did not differ significantly from those obtained with the positive control (P > 0.05). The EC_50_ value estimated for the EO in the EHT was 0.19 mg/mL.

**Table 2:** Efficacy (mean ± standard deviation) of *Ocimum micranthum* essential oil on the hatching of *Haemonchus contortus* eggs.

**Concentration (mg/mL)**	**Efficacy**
**1**	100 (± 0.00)^A^
**0.50**	98.01 (± 1.33)^A^
**0.25**	77.25 (± 7.12)^B^
**0.12**	30.23 (± 9.23)^C^
**0.06**	7.62 (± 4.21)^D^
**DMSO (0.5%)** ^*^	1.28 (± 0.95)^E^
**Thiabendazole (0.025 mg/mL)** ^**^	96.34 (± 2.70)^A^

* Negative control;

** Positive control. The capital letters indicate comparisons of the means in the columns. Different letters indicate significantly different values (P < 0.05).

The LDT results are presented in Table 3. At concentrations of 0.12 and 0.06 mg/mL, the EO inhibited larval development by 100% and 97.06%, respectively, with no significant difference relative to the positive control. At the lowest concentration evaluated (0.007 mg/mL), the EO showed no significant effect compared with the negative control (P > 0.05). The EC_50_ value for inhibition of larval development was 0.031 mg/mL, indicating a concentration-dependent effect.

**Table 3:** Efficacy (mean ± standard deviation) of *Ocimum micranthum* essential oil on the development of *Haemonchus contortus* larvae.

**Concentration (mg/mL)**	**Efficacy**
**0.12**	100.00 (± 0.00)^A^
**0.06**	97.06 (± 1.07)^A^
**0.03**	44.63 (± 6.70)^B^
**0.015**	20.20 (± 3.87)^C^
**0.007**	5.27 (± 7.15)^D^
**DMSO (0.5%)** ^*^	0.18 (± 0.40)^D^
**Ivermectin (0.008 mg/mL)** ^**^	100.00 (± 0.00)^A^

* Negative control;

** Positive control. The capital letters indicate comparisons of the means in the columns. Different letters indicate significantly different values (P < 0.05).

The inhibitory effects of *O. micranthum* EO on larval migration is presented in Table 4. The EO demonstrated larvicidal activity at all concentrations tested, also in a concentration-dependent manner. Larval migration was inhibited by more than 75% at concentrations of 4, 2, and 1 mg/ml, with no significant differences compared with the positive control (P > 0.05). The EC_50_ value calculated for this assay was 0.63 mg/mL.

**Table 4:** Efficacy (mean ± standard deviation) of *Ocimum micranthum* essential oil on the migration of *Haemonchus contortus* larvae.

**Concentration (mg/mL)**	**Efficacy**
**4**	85.63 (± 3.66)^A^
**2**	79.18 (± 5.78)^A^
**1**	76.22 (± 6.66)^A^
**0.50**	60.01 (± 9.64)^B^
**0.25**	12.26 (± 12.94)^C^
**DMSO (0.5%)** ^*^	3.57 (± 5.33)^D^
**Ivermectin (0.25 mg/mL)** ^**^	91.41 (± 5.58)^A^

* Negative control;

** Positive control. The capital letters indicate comparisons of the means in the columns. Different letters indicate significantly different values (P < 0.05).

In the AWMT, *O. micranthum* EO at 0.25 mg/mL completely inhibited the motility of adult *H. contortus* from the first evaluated time point (3 h post-exposure). At concentrations of 0.25 and 0.125 mg/mL, the EO exhibited efficacy comparable to ivermectin, achieving 100% inhibition after 9 and 12 h of incubation (P > 0.05). Furthermore, after 12 h of exposure, more than 50% inhibition of adult motility was observed at a concentration of 0.03 mg/mL. The EC_50_ value estimated after 12 h of incubation was 0.046 mg/mL, demonstrating a concentration-dependent effect. These data are summarized in Table 5.

**Table 5:** Efficacy (mean ± standard deviation) of *Ocimum micranthum* essential oil on the motility inhibition of adult *Haemonchus contortus*.

**Concentrations (mg/mL)**	**Exposure time of adult worms to treatment (hours)**			
	**3h**	**6h**	**9h**	**12h**
0.25	100.00 ± 0.00^Aa^	100.00 ± 0.00^Aa^	100.00 ± 0.00^Aa^	100.00 ± 0.00^Aa^
0.12	46.67 ± 18.26^Ba^	93.33 ± 14.91^Ab^	100.00 ± 0.00^Ab^	100.00 ± 0.00^Ab^
0.06	33.33 ± 0.00^Ba^	40.00 ± 14.91^Ba^	53.33 ± 18.26^Ba^	60.00 ± 27.89^Ba^
0.03	0.00 ± 0.00^Ca^	0.00 ± 0.00^Ca^	0.00 ± 0.00^Ca^	53.33 ± 18.26^Bb^
0.015	0.00 ± 0.00^Ca^	0.00 ± 0.00^Ca^	0.00 ± 0.00^Ca^	0.00 ± 0.00^Ca^
Ivermectin (0.10 mg/mL)^*^	60.02 ± 14.91^Da^	66.67 ± 23.57^Da^	100.00 ± 0.00^Ab^	100.00 ± 0.00^Ab^
DMSO (0.5%)^**^	0.00 ± 0.00^Ca^	0.00 ± 0.00^Ca^	0.00 ± 0.00^Ca^	0.00 ± 0.00^Ca^

* Positive control;

** Negative control. The capital letters indicate comparisons of the means in the columns, and the lowercase letters denote comparisons of the means in the rows. Different letters indicate significantly different values (P < 0.05).

**Figure 1:**
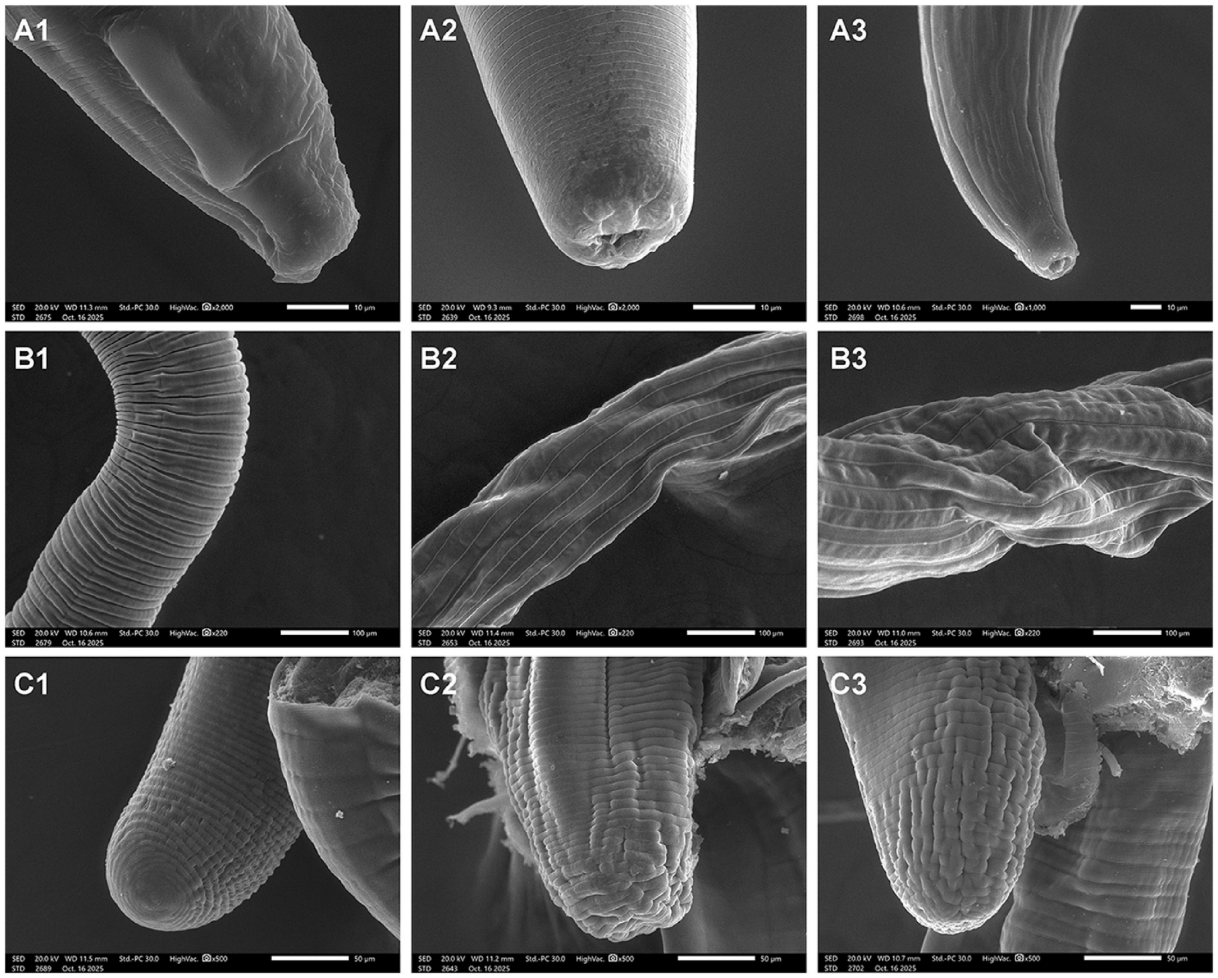
Scanning electron microscopy of adult *Haemonchus contortus* incubated in 0.5% DMSO (A1, B1 and C1), 0.25 mg/mL *Ocimum micranthum* essential oil (A2, B2 and C2), and 0.10 mg/mL ivermectin (A3, B3 and C3). Scale bars: 10 μm (A1–A3), 100 μm (B1–B3), and 50 μm (C1–C3).

The morphological alterations observed in adult females *H. contortus* following *in vitro* exposure to the EO and controls are illustrated in Figure 1. SEM revealed that untreated adult females (negative control) displayed a preserved cephalic region, intact longitudinal cuticular ridges, and vulvar flap without visible alterations (Figure 1–A1, B1, C1). In contrast, females exposed to *O. micranthum* EO (0.25 mg/mL) displayed surface damage, including retraction of the buccal capsule with lancet loss in the cephalic region (Figure 1–A2) as well as wrinkling and thickening of the cuticle and vulvar flap (Figure 1–B2, C2). These alterations were similar to those observed in worms exposed to ivermectin (positive control; Figure 1–A3, B3, C3).

**Figure 2:**
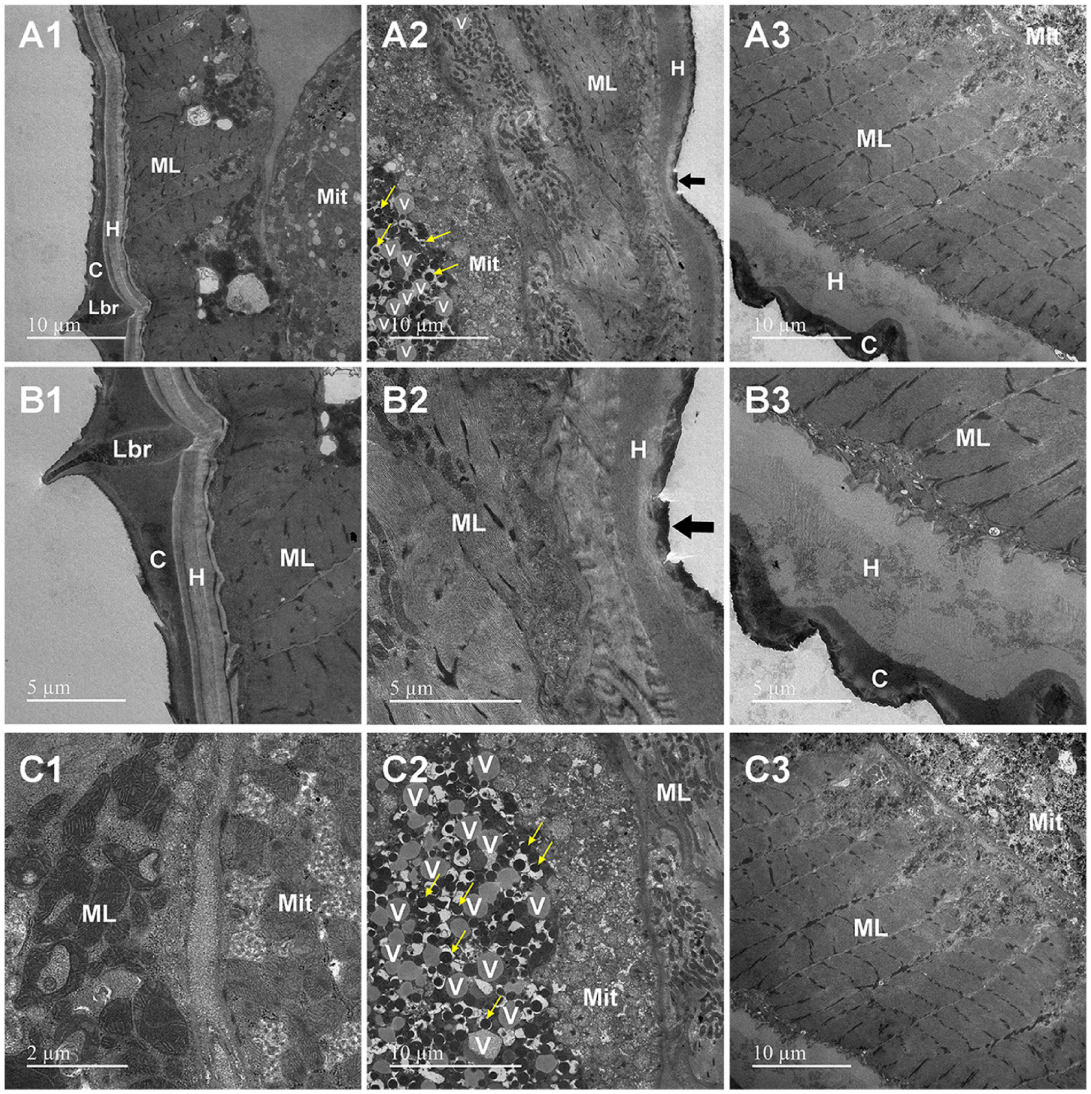
Transmission electron microscopy of adult *Haemonchus contortus* incubated in 0.5% DMSO (A1, B1 and C1), 0.25 mg/mL *Ocimum micranthum* essential oil (A2, B2 and C2), and 0.10 mg/mL ivermectin (A3, B3 and C3). Structures shown: cuticle (C), hypodermis (H), muscle layer (ML), longitudinal body ridge (Lbr), mitochondria (Mit), and vacuole (V). Black arrows indicate cuticle thickening; yellow arrows mark intracellular granular material suggestive of lysis after essential oil exposure. Scale bars: 10 μm (A1–A3, C2-C3), 5 μm (B1–B3), and 2 μm (C1).

In TEM micrographs, the untreated (negative control) *H. contortus* adults of the AWMT exhibited a typical internal morphology (Figure 2–A1, B1, C1). In contrast, adults exposed to *O. micranthum* EO showed ultrastructural modifications. The cuticle appeared thickened and displayed loss of regular organization. Alterations were also observed in the muscular layer including fiber disorganization and the presence of vacuoles, along with signs of cytoplasmic degeneration. Mitochondria showed altered morphology, with vacuoles containing granular intracellular material, suggestive of lysis (Figure 2–A2, B2, C2). Adult nematodes exposed to ivermectin (positive control) exhibited extensive ultrastructural alterations, including cuticular degradation with rupture sites and irregular contours. In the muscular layer, fibers were completely disrupted, and mitochondria exhibited severe alterations, including destruction of structural integrity, indicative of membrane collapse (Figure 2–A3, B3, C3).

## Discussion

The use of plant-derived natural products as alternatives for controlling GIN in small ruminants has been extensively investigated. To our knowledge, this is the first study to evaluate the anthelmintic potential of *O. micranthum* EO against *H. contortus* and reports its activity against the eggs, larvae, and adults of a multidrug-resistant isolate.

Chemical analysis identified 20 compounds in *O. micranthum* EO, with eugenol accounting for more than 70% of the oil composition. This finding is consistent with previous studies reporting eugenol as the major component of *O. micranthum* EO cultivated in Brazil and Ecuador (Silva et al., 1998; Sacchetti et al., 2004; Barbosa, 2018; Freitas et al., 2018; Scalvenzi et al., 2019; Ricarte et al., 2020; Tacchini et al., 2021; Guerrini et al., 2023). In contrast, methyl cinnamate, methyl eugenol, caryophyllene, and eucalyptol have been reported as major constituents in plants collected in Peru, Mexico, Colombia, and Brazil (Benitez et al., 2009; Pinho et al., 2012; Silva et al., 2014; Caamal-Herrera et al., 2016; Wilson et al., 2022; Calvo‐Irabien, 2025). Variations in EOs composition represent a major limitation for their use as anthelmintic therapies and may be influenced by environmental conditions, plant parts, developmental stage, harvest season, processing and storage conditions (Bakkali et al., 2008; Amel et al., 2024).

EO obtained from *Syzygium aromaticum* (clove), mainly composed of eugenol, has shown acaricidal activity against different life stages of *Rhipicephalus sanguineus*, *Rhipicephalus microplus*, and *Hyalomma scupense* (Ferreira et al., 2018a; Alimi et al., 2023; Abdel-Ghany et al., 2026). Eugenol was considered the main compound responsible for the clove EO acaricidal properties (Abdel-Ghany et al., 2026). The anthelmintic activity of eugenol has been reported against *Schistosoma mansoni* (El-kady et al., 2019), *Echinococcus granulosus* (Maurice et al., 2021), *Trichinella spiralis* (ElGhannam et al., 2023), and *H. contortus* (Oliveira et al., 2026). This phenolic compound contains a hydroxyl group that may facilitate interactions with biological targets (Oliveira et al., 2026).

The ovicidal activity of *O. gratissimum* and *O. basilicum* EOs extracted in Brazil has previously been evaluated against *H. contortus*. At the highest concentrations tested (1, 0.50, and 0.25%), the *O. gratissimum* efficacy exceeded 90% (Pessoa et al., 2002). This result is comparable to that obtained in our study with *O. micranthum* EO, which also showed efficacy greater than 90%. The EC_50_ values of cultivars *Ocimum basilicum* ranging from 0.56 to 2.22 mg/mL (Sousa et al., 2021). In contrast, in the present study, *O. micranthum* EO demonstrated an EC_50_ of 0.19 mg/mL in the EHT. In addition, we demonstrated that the efficacy of the EO was higher than that of eugenol, as reported in other studies (Katiki et al., 2017; Sousa et al., 2021). Katiki et al. (2017) and Sousa et al. (2021) demonstrated EC_50_ values of 0.57 and 1.39 mg/mL, respectively, for eugenol. The biological activity of EOs is often attributed to their major constituents; however, these oils may contain up to 40 components with additive or synergistic effects (Bakkali et al., 2008; Katiki et al., 2017).

Other EOs obtained from Lamiaceae species have also demonstrated activity against *H. contortus* larvae. However, they showed a lower larvicidal efficacy than *O. micranthum*. In LDT, the EC_50_ values of *Hesperozygis myrtoides* and *Lavandula officinalis* EOs were 0.072 and 0.280 mg/mL, respectively (Castilho et al., 2017; Ferreira et al., 2018b). Nevertheless, the effect of *O. micranthum* EO against larvae was inferior to that of *Thymus vulgaris* EO and its major component, thymol, which exhibited EC_50_ values of 0.013 and 0.022 mg/mL, respectively (Ferreira et al., 2016). The aqueous and methanolic extracts of *O. sanctum* leaves have been evaluated for their effects on *H. contortus* larval development, with EC_50_ values of 7.746 and 28.199 mg/mL, respectively (Kanojiya et al., 2015). In comparison, *O. micranthum* EO (EC_50_: 0.031 mg/mL) demonstrated superior activity against larvae relative to *O. sanctum* extracts.

Nematodes are covered by a resistant, semipermeable external layer known as the cuticle. This structure is replaced at each molt; however, infective *H. contortus* L3 retains the cuticle of the second-stage larvae resulting in a double cuticle that protects the parasite and increases environmental resistance (Zajac & Garza, 2020). Therefore, evaluating anthelmintic activity against L3 is particularly important. Castro et al. (2021) and Garbin et al. (2025) assessed the effects of *Anethum graveolens* and *Citrus aurantium bergamia* EO on L3 migration inhibition, reporting EC_50_ values of 3.963 and 24.47 mg/mL, respectively. The anthelmintic activity of *Mentha villosa* and *Mentha × piperita* EO, as well as their major metabolites carvone and limonene, on larval migration inhibition also was evaluated, yielding EC_50_ values of 3.04, 4.23, 1.96, and 14.27 mg/mL, respectively (Bortoluzzi et al., 2021). These values were higher than those obtained in our study with *O. micranthum* EO (EC_50_ of 0.63 mg/mL), indicating a higher inhibitory effect of this oil against L3.

The anthelmintic activity of *O. micranthum* EO against the different life stages of *H. contortus* may be associated with the lipophilic nature of its hydrocarbon backbone and the hydrophilic nature of its functional groups, as previously reported for species of the genus *Candida* (Vieira et al., 2014; Caamal-Herrera et al., 2018). EOs rich in phenolic compounds, such as *O. micranthum* oil, tend to exhibit higher activity against microorganisms and a broader spectrum of action. This is attributed to the acidic nature of the hydroxyl group of eugenol that enables hydrogen bond formation with the active site of enzymes (Vieira et al., 2014).

Studies on the anthelmintic activity of EOs against adult GIN are particularly relevant, because this life stage represents the primary target of synthetic anthelmintics. In the AWMT, *O. micranthum* EO (0.25 mg/mL) completely inhibited the motility of adult females from the first observation time and inhibited more than 50% of worms at a concentration of 0.03 mg/mL after 12 h of incubation. Our findings are consistent with those of previous studies that investigated the potential of EO from the Lamiaceae family, which are rich in phenolic compounds. Ferreira et al. (2016) evaluated the activity of *T. vulgaris* EO (50, 5, and 0.5 mg/mL) and its major compound thymol (25, 2.5, and 0.25 mg/mL), and all concentrations tested completely inhibited adult motility within the first 8 h of the experiment. Similarly, *L. officinalis* EO (50, 5, and 0.5 mg/mL) inhibited 100% of adult *H. contortus* motility after 12 h of exposure (Ferreira et al., 2018b). Amel et al. (2024) demonstrated that *T. capitatus* EO (1 mg/mL) inhibits the motility of adult nematodes by 70.8% after 8 h of incubation. These results reinforce the anthelmintic potential of EOs, which stand out as environmentally degradable and effective alternatives to multidrug-resistant GIN.

The present study demonstrated that treatment with *O. micranthum* EO induces marked morphological and ultrastructural alterations in adult *H. contortus*. SEM images revealed, as the main changes, retraction of the buccal capsule with loss of the lancet in the cephalic region, as well as wrinkling and thickening of the cuticle and the vulvar flap of the nematode. The cuticle is responsible for maintaining worm body shape and, within the host gastrointestinal tract, plays a fundamental role in motility and interactions with the parasitic environment, including metabolic interactions (Mohamed et al., 2024). Thus, cuticular damage may impair free nematode movement, interfering with the search for feeding sites and mating opportunities (Martínez-Ortíz-de-Montellano et al., 2013). According to Stepek et al. (2010), the damage in nematodes cuticular surface become more susceptible to the host's immune response.

Damage to the buccal capsule may disrupt the mechanical and/or enzymatic processes involved in blood ingestion, potentially resulting in malnutrition, reduced fertility, and/or parasite mortality. Additionally, vulvar flap thickening was observed, which may negatively affect the reproductive function of females either by mechanically obstructing egg production or by expulsion (Martínez-Ortíz-de-Montellano et al., 2013). Therefore, the findings of the present study suggest that treatment with the oil induces morphological damage to the structure of the worms, which likely contributes to the observed physiological impairment and mortality.

TEM-based studies investigating ultrastructural damage in *H. contortus* induced by natural products, including plant extracts (Brunet et al., 2011; Martínez-Ortiz-de-Montellano et al., 2019; Rocha et al., 2020), EOs, and isolated metabolites (Araújo-Filho et al., 2019), indicate that loss of motility is frequently associated with tegumentary and muscular damage, such as disruption of the hypodermis with the cuticle, vacuolization of muscle cells, and disorganization of muscle fibers, accompanied by mitochondrial alterations characterized by the absence of matrix and cristae. In the present study, TEM images revealed marked ultrastructural alterations in *H. contortus* exposed to *O. micranthum* EO, including cuticle thickening, disorganization of the muscle layer, vacuole formation, and changes in the mitochondrial profile, with the presence of vacuoles containing granular intracellular material. These findings were consistent with those of previous studies (Brunet et al., 2011; Araújo-Filho et al., 2019; Martínez-Ortiz-de-Montellano et al., 2019; Rocha et al., 2020). Furthermore, the presence of vacuoles and granular intracellular material indicates severe impairment of cellular integrity. These alterations suggest degeneration and/or death of muscle cells and mitochondria that are essential structures for the maintenance of cellular homeostasis and energy metabolism (Brunet et al., 2011) and may therefore contribute to the worm mortality observed in the present study.

## Conclusion

*Ocimum micranthum* EO exhibited *in vitro* activity against all life stages of *H. contortus*. Moreover, morphological and ultrastructural alterations were observed in adult parasites, suggesting that the oil compromised their structural integrity, ultimately resulting in motility inhibition. These findings underscore the potential of this natural product as a sustainable alternative to synthetic anthelmintics, given its possible synergistic phytochemical properties and multiple mechanisms of action. However, further studies to evaluate *in vivo* efficacy, toxicological safety, and pharmacokinetic profile are required to validate its therapeutic potential.

## Data Availability

Research data is only available upon request.
